# Reducing Inflammation and Vascular Invasion in Intervertebral Disc Degeneration *via* Cystathionine-γ-Lyase Inhibitory Effect on E-Selectin

**DOI:** 10.3389/fcell.2021.741046

**Published:** 2021-11-15

**Authors:** Haoran Xu, Kang Wei, Jingyao Tu, Yangmengfan Chen, Yi He, Yifan Ding, Huanhuan Xu, Xinyu Bao, Hui Xie, Huang Fang, Huan Wang

**Affiliations:** ^1^Department of Orthopedics, Tongji Hospital, Tongji Medical College, Huazhong University of Science and Technology, Wuhan, China; ^2^Department of Oncology, Tongji Hospital, Tongji Medical College, Huazhong University of Science and Technology, Wuhan, China; ^3^Department of Obstetrics and Gynecology, Wuhan Children’s Hospital, Tongji Medical College, Huazhong University of Science and Technology, Wuhan, China; ^4^School of Medicine and Health Management, Tongji Medical College, Huazhong University of Science and Technology, Wuhan, China; ^5^Department of Radiology, Tongji Hospital, Tongji Medical College, Huazhong University of Science and Technology, Wuhan, China

**Keywords:** intervertebral disc degeneration (IDD), inflammation, vascular invasion, cystathionine-γ-lyase (CSE), E-selectin (CD62E)

## Abstract

The incidence of degenerative spinal diseases, such as cervical spondylosis and thoracic and lumbar disc herniation, is increasing. These health problems have adversely affected human life and work. Surgical intervention is effective when intervertebral disc degeneration (IDD) causes nerve compression and/or severely limits daily activity. Early IDD patients generally do not require surgery. However, there is no effective method of impeding IDD progression. Thus, novel approaches to alleviating IDD deterioration are urgently required. Cystathionine-γ-lyase (CSE) and E-selectin (CD62E) are vital factors regulating vascular function and inflammation. However, their effects on IDD and vascular invasion in intervertebral discs (IVDs) are pending further exploration. Here, bioinformatics and human nucleus pulposus (NP) tissues analyses revealed that CSE was significantly downregulated and CD62E was upregulated in the NP tissues of IDD patients. We demonstrated that CSE overexpression, CD62E downregulation, and NF-κB (P65) inhibition mitigate inflammation and recover metabolic function in NP cells. Similarly, CSE attenuated vascular invasion induced by inflammatory irritation. Using a rat IDD model, we showed that CSE improved degeneration, inflammation, and microvascular invasion in NP tissue, whereas CD62E had the opposite effect. Taken together, our results indicated that the CSE/CD62E pathway could effectively improve the inflammatory environment and vascular invasion in IVD. Hence, the findings of this study propose a promising and valuable strategy for the treatment of patients with early IDD as well as postoperative adjuvant therapy in patients with severe IDD.

## Introduction

Degenerative spinal diseases are associated with a series of chronic and progressive symptoms and can have a tremendous impact on the quality of human life (Khan et al., 2017; Molladavoodi et al., 2020; Yang et al., 2020). The incidence and early onset of degenerative spinal disease are increasing because of a growing aging population and an unhealthy lifestyle, respectively (Salminen et al., 1999; Karademir et al., 2017). Intervertebral disc degeneration (IDD) causes low back and leg pain and may compress spinal nerves in severe cases. However, obvious clinical symptoms do not always manifest in early IDD patients. Therefore, conservative treatments must be administered to alleviate certain clinical symptoms. Recently, a few studies reported that conservative treatments alone cannot attenuate the inflammatory environment or recover normal functioning of the nucleus pulposus (NP) in degenerating intervertebral discs (IVDs). Thus, IDD might recur or worsen (Sharifi et al., 2015; Zhao et al., 2019). Consequently, innovative therapies are required for IDD.

The pathogenesis of IDD mainly about metabolic dysfunction occurs in the extracellular matrix (ECM) of the IVD and the NP cells undergo apoptosis (Blanquer et al., 2015; Vergroesen et al., 2015). Several researchers investigated the risk factors associated with inflammatory irritation and recognized it as one of the major factors leading to ECM decomposition, NP cell apoptosis, and IDD pathogenesis (Feng et al., 2016; Khan et al., 2017; Ruiz-Fernández et al., 2019; Cazzanelli and Wuertz-Kozak, 2020). It was reported that the activation of inflammatory-related pathways, such as NF-κB, Akt/PI3K, and MAPK, increases the production and secretion of apoptotic proteins and proinflammatory ILs, TNF, and MMPs. These responses induce NP cell apoptosis and fibrotic matrix biosynthesis (Sakai and Grad, 2015). Microvessel invasion and angiogenesis in the IVD also aggravate IDD (Fontana et al., 2015; Sakai and Grad, 2015; Vergroesen et al., 2015; Oichi et al., 2020). Under normal physiological conditions, the function and hypoxic status of the IVD are regulated and maintained by the vascular tissue in the endplate cartilage (Dowdell et al., 2017). During IVD degeneration, NP protrusion and/or annulus fibrosus (AF) disruption may induce vascularization and innervation (Freemont et al., 2002; Oichi et al., 2020). These changes recruit immune cells and inflammatory factors into the IVD (Sun et al., 2020) and cause oxidative stress. Therefore, IVD inflammatory stimulation and vascularization create a vicious cycle aggravating IDD.

In the attempt to break this vicious circle, we sought new targets that can mitigate inflammation and angiogenesis in the IVD. A bioinformatics analysis of the differences between patients with adolescent idiopathic scoliosis and those with IDD disclosed that cystathionine-γ-lyase (CSE) was significantly downregulated in the IDD group compared to the normal (control) group. Recent published evidence revealed that CSE regulates vessel function and eliminates inflammation (Yang et al., 2008; Castelblanco et al., 2018; Sun et al., 2019; Éva Sikura et al., 2021). Nevertheless, the roles of CSE in IDD have not been explored. CD62E is a key inflammation mediator (Del Bo et al., 2019; Milošević et al., 2020) and might be regulated by CSE (Bibli et al., 2019). It may activate the NF-κB pathway, thereby exacerbating IVD degeneration (Tisherman et al., 2016; Chen et al., 2020a, b). Moreover, the vascular effects of inflammatory pathway activation also accelerate IDD deterioration (Wang et al., 2020). Therefore, the CSE regulatory effect on inflammation and vascular function is a potential mechanism of restricting IDD progression. It is useful to examine the relationship between CSE and CD62E to identify new targets for improving IDD.

Despite numerous research efforts in recent decades, the mechanisms of IDD have not been fully elucidated and few effective treatments for it have been identified. Our histochemical and bioinformatics analyses of NP tissues in patients with IDD have indicated that IDD progression is regulated by CSE, CD62E, and the NF-κB pathway. We hypothesized that downregulating CD62E with CSE and blocking downstream inflammatory pathways could effectively improve inflammation and vascular invasion in IDD. To test our hypothesis, we performed various *in vitro* and *in vivo* experiments to clarify the relationships among CSE, CD62E, and the NF-κB and VEGF-A pathways and establish their roles in IDD progression. The results of the study could lead to the discovery of novel targets for alleviating IDD as well as potential strategies for impeding IDD progression.

## Materials and Methods

### Experimental Materials and Reagents

Recombinant human interleukin-1 beta (IL-1β), tumor necrosis factor-alpha (TNF-α), caffeic acid phenethyl ester (CAPE), and ICAM-1-IN-1 were purchased from MedChemExpress LLC, Monmouth Junction, NJ, United States. Cell Counting Kit-8 (CCK-8) was bought from Vazyme Biotech, Nanjing, China. Growth factor-reduced matrigel matrix (354234) was purchased from BD Biosciences, Franklin Lakes, NJ, United States. CSE overexpression adenovirus (AV-CSE) and CD62E overexpression adenovirus (AV-CD62E) were constructed by WZ Bioscience Inc., Wuhan, China.

### Animals

Male Sprague–Dawley (SD) rats (6–8 weeks; 220 ± 20 g) were obtained from the Disease Control Center of Hubei, China. All animal experiments were conducted in specific pathogen-free (SPF) conditions at Tongji Animal Center (Wuhan, China) according to the International Guiding Principles for Animal Research and in compliance with regulations and guidelines of the Ethics Committee of Huazhong University of Science and Technology, Wuhan, China.

### Cell Culture

Nucleus pulposus cells were harvested from the IVD inner tissue of SD rats (200–220 g). NP tissues were cut, separated, and digested with 0.25% trypsin at 37°C for 30 min and with type II collagenase at 37°C for 3 h. The cells were collected and cultured in DMEM/F12 supplemented with 10% fetal bovine serum (Gibco, Grand Island, NY, United States) and 1% penicillin/streptomycin (Gibco) in a 5% CO_2_ incubator at 37°C. Endothelial progenitor cells (EPCs) were obtained from the Chinese Academy of Sciences Cells Bank (Shanghai, China) and cultured under the foregoing conditions.

### Human Nucleus Pulposus Tissue Collection

Human NP tissues were harvested from patients who had undergone surgery at the Department of Orthopedics of Tongji Hospital, Wuhan, China. The degenerated NP tissue was derived from IDD patients and the normal NP tissue was collected from patients with scoliosis or spinal fracture. During the operation, the isolated NP tissue was washed thrice with saline and adjacent non-NP tissue was removed. The NP tissue was either fixed with 4% paraformaldehyde (PFA) or the protein in it was extracted for further experimentation. This process was conducted with patient consent and reviewed by the Ethics Committee of Huazhong University of Science and Technology, Wuhan, China.

### Cell Viability Assay

To evaluate their post-treatment viability, NP cells and EPCs were seeded into 96-well plates (5 × 10^3^/well) and there were three replicate wells per group. After 24 h, the cells were subjected to IL-1β (20 ng/mL), TNF-α (20 ng/mL), CAPE (1 μM), ICAM-1-IN-1 (1 μM), and AV-CSE (MOI = 100) for 24 or 48 h. CCK-8 was used to evaluate cell viability, and the absorbance of each well was measured at 450 nm.

### Bioinformatics Analysis

A microarray dataset (accession no. GSE34095) was obtained from the GEO database.^1^ This microarray dataset comes from the research “Expression data between human degenerative and non-degenerative intervertebral discs.” It provided valuable insight into the molecular mechanism of IDD and facilitated this research. Differentially expressed genes (DEGs) were those with *p* < 0.05 and |log2 (Fold Change)| > 1. They were depicted in a heat map and a volcano plot. A Gene Ontology (GO) analysis of the top genes was performed using DAVID Bioinformatics Resources.

### Western Blot Analysis

Nucleus pulposus tissues or cells were homogenized in RIPA buffer containing phosphatase and protease inhibitors. Total proteins were collected and extracted by centrifugation at 12,000 rpm and 4°C for 30 min. A BCA Protein Assay Kit (Beyotime Biotechnology, Shanghai, China) was used to evaluate the protein concentration. Thirty micrograms of total proteins was separated by sodium dodecyl sulfate polyacrylamide gel electrophoresis (SDS-PAGE) and electrotransferred to a 0.45-μm PVDF membrane (Millipore, Billerica, MA, United States), blocked with 5% BSA for 1 h, and incubated with primary antibodies overnight at 4°C. The membrane was then immunoblotted with the corresponding secondary antibody and visualized with the SuperSignal West Pico chemiluminescent substrate (Thermo Fisher Scientific, Waltham, MA, United States). The blots were analyzed with a Chemidoc XRS imaging system (Bio-Rad Laboratories, Hercules, CA, United States). The major antibodies used were GAPDH (60004-1-lg, 1:5000, Proteintech, Wuhan, China), CSE (12217-1-AP, 1:1000, Proteintech), CD62E (20894-1-AP, 1:1000, Proteintech), NF-κB P65 (#8242, 1:1000, Cell Signaling Technology, Boston, MA, United States), phospho-NF-κB (#3033, 1:1000, Cell Signaling Technology), VEGF-A (ab46154, 1:1000, Abcam, Cambridge, United Kingdom), MMP-3 (66338-1-lg, 1:1000, Proteintech), MMP-9 (10375-2-AP, 1:1000, Proteintech), MMP-13 (18165-1-AP, 1:1000, Proteintech), COL1A1 (#84336, 1:1000, Cell Signaling Technology), and COL2A1 (28459-1-AP, 1:1000, Proteintech).

### RT-PCR Analysis

Total RNA was extracted with RNAiso Plus (TaKaRa Bio Inc., Kusatsu, Shiga, Japan) and reverse-transcribed to cDNA with reverse transcriptase (Toyobo, Osaka, Japan). The cDNA was amplified with specific primers and SYBR Premix Ex Tap (Tli RNaseH Plus) (2×) (Toyobo). Primer sequences are shown in Supplementary Table 1. The real-time PCR detection system (Bio-Rad Laboratories) was used to measure fluorescence. Target gene mRNA expression levels were quantified by the 2^–ΔΔ*Ct*^ method.

### Tube Formation Assay

Growth factor-reduced matrigel matrix (10 mg/mL) was added to a 96-well plate (50 μL per well) and incubated at 37°C for 30 min. After solidification, EPCs (1.0 × 10^4^/well) were seeded into matrigel surfaces containing the various treatments and incubated at 37°C. Each well image was captured at 0, 3, and 6 h and stained with calcein-AM at the end of the experiment. There were three images per well and the magnifications used were 100× and 200×. The tube-like structures were enumerated and calculated with ImageJ (NIH, Bethesda, MD, United States).

### Transwell Migration Assay

A 6.5-mm 24-well transwell plate with the 8.0-μm pore polycarbonate membrane insert (3422, Corning, NY, United States) was used in this assay. Serum-containing DMEM/F12 (10%) was added to the lower chamber. EPCs (2.0 × 10^4^/well) were seeded into the upper chamber containing the serum-free medium. After 24 h incubation, non-migrating cells on the upper chamber side were scraped with cotton swabs. Migrating cells on the lower membrane sides were fixed with 4% PFA and stained with 0.1% crystal violet dye. Stained cells were enumerated and analyzed with ImageJ (NIH).

### Immunohistochemistry Assay

Isolated NP tissues were fixed with 4% PFA for 24 h and decalcified with the EDTA solution for 1 month. The tissues were dehydrated, embedded in paraffin, sectioned, and mounted on slides. Hematoxylin–eosin (H&E) staining (Servicebio, Wuhan, China), Safranin O/Fast Green staining (Servicebio), and immunohistochemistry (IHC) staining assays were conducted according to the manufacturers’ protocols. The foregoing major antibodies were used, and they were diluted to 1:200. Bright-field images were captured with a microscope (ECLIPSE Ti; Nikon, Tokyo, Japan). The IHC staining was digitally quantitated with ImageJ (NIH).

### Immunofluorescence Analysis

Nucleus pulposus cells were cultured in a 48-well plate (1 × 10^4^/well) and incubated at 37°C. After cell attachment, the various treatments were applied to the cells for 24 h. Thirty minutes before fixation with 4% PFA, the cells were stimulated by adding the proinflammatory factors IL-1β and TNF-α to the culture medium. The cells were fixed, permeabilized with 0.1% Triton X-100, and blocked with 0.1% BSA according to the manufacturers’ protocols. NF-κB antibody (#8242, 1:200, Cell Signaling Technology) and phospho-NF-κB (#3033, 1:500, Cell Signaling Technology) were used to detect inflammatory changes. Freshly dissected NP tissues were fixed in 4% PFA for 24 h and embedded in paraffin. The vascular marker CD31 antibody (A11525, 1:200, ABclonal Technology, Woburn, MA, United States) was used to display blood vessel architecture and vascular invasion. Immunofluorescence (IF) images were observed and captured by fluorescence microscopy (EVOS FL auto, Thermo Fisher Scientific) and analyzed by ImageJ (NIH).

### Magnetic Resonance Imaging

Each rat body was set in the prone position with tail straight under a 1.5-TMR scanner (Siemens, Berlin, Germany). The rats were maintained under 2% isoflurane/oxygen anesthesia throughout the examination. Eight consecutive sagittal T2-weighted images were obtained by scanning the surgical regions of the rats with a double-tuned volume radiofrequency coil. All vertebral disc regions were covered with a spin-echo sequence (Wei et al., 2020). The magnetic resonance imaging (MRI) images were analyzed by the Pfirrmann score (Pfirrmann et al., 2001).

### Animal Model Generation and Treatment

An IDD model can be induced *via* needle puncture to a rat tail disc (Vergroesen et al., 2015; Mosley et al., 2019). The rats were anesthetized with isoflurane, disinfected with iodophor, and placed in a sterile surgical area. The intervertebral disc level was confirmed by palpation and a longitudinal incision (∼2 mm) was made on the tail skin and the tendon to expose the surgical field of the NP (Supplementary Figure 2B). The experimental animals were divided into four groups (*n* = 12) as follows: (1) The Sham group received no treatment and their incisions were sutured. (2) The NP tissues of the GFP group were injected with GFP adenovirus (AV-GFP) with a 31-gauge Hamilton syringe (Hamilton Co., Reno, NV, United States) and their incisions were sutured. (3) The NP tissues of the IDD + AV-GFP group were punctured with an 18-gauge (O.D.) needle for 10 s injected with AV-GFP and their incisions were sutured. (4) The NP tissues of the IDD + AV-CSE group were punctured and injected with AV-CSE as previously described. To determine the influences of AV-CD62E on IDD onset, the NP tissues were injected with saline (Sham), AV-GFP, or AV-CD62E (*n* = 12). The IVD models were then subjected to MRI and IHC at 2 and 4 weeks after surgery.

### Statistical Analysis

Statistical analysis of all data was performed in GraphPad Prism 8 (GraphPad Software, La Jolla, CA, United States). Student’s *t*-test was used to compare group means, and one-way ANOVA (analysis of variance) was used to compare multiple samples. Data were presented as means ± standard deviation (SD). A Kruskal–Wallis *H* test was used to analyze the Pfirrmann score. Data were presented as medians with interquartile ranges and were ordinal variables. Statistical significance was considered as *p < 0.05, **p < 0.01, and ***p < 0.001.

## Results

### Roles of Cystathionine-γ-Lyase and CD62E in Intervertebral Disc Degeneration

**FIGURE 1:**
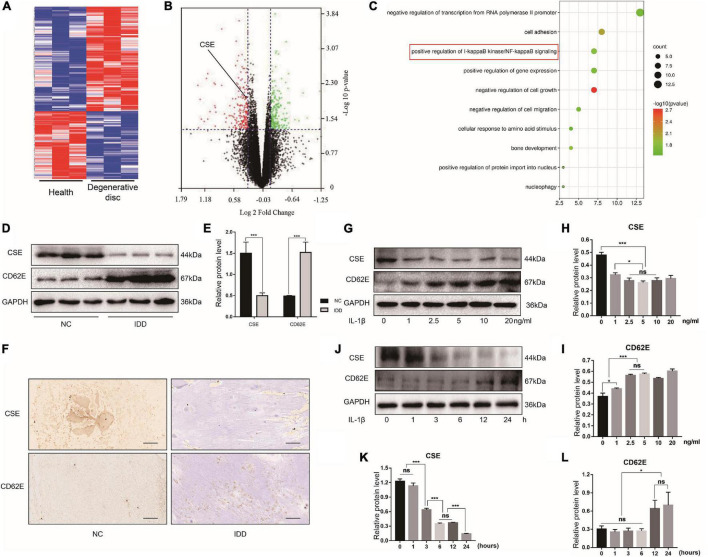
Cystathionine-γ-lyase and CD62E expression in NP tissues and cells. Bioinformatics analysis of expression profiles of patients with IDD and adolescent idiopathic scoliosis. **(A)** Heat map of top 300 DEGs. **(B)** Volcano plot of DEGs shows significantly altered mRNA expression with *p* < 0.05 (green dots: upregulated DEGs; red dots: downregulated DEGs). **(C)** GO analysis term. **(D)** Western blot images of CSE and CD62E and **(E)** its quantitative analysis. **(F)** IHC images of NP tissue (CSE and CD62E). **(G)** Representative western blot images of NP cells after stimulation with various doses of IL-1β for 24 h and **(H,I)** its quantitative analysis. **(J)** Representative western blot images of NP cells after stimulation with 5 ng/mL IL-1β for different times and **(K,L)** its quantitative analysis. Black scale bar: 100 μm.

We conducted bioinformatics analyses of the gene expression profiles of patients with the degenerative disc disease and adolescent idiopathic scoliosis to determine the functions of CSE in intervertebral disc maintenance (Figure 1A). The identified DEGs revealed that CSE was significantly downregulated in IDD patients (Figure 1B) and closely associated with NF-κB pathway activation (Figure 1C). Human NP tissue western blot and IHC analyses disclosed that CSE was upregulated in patients with scoliosis and spinal fracture (NC group) compared to those with IDD and spinal fusion (IDD group). However, the CD62E expression pattern was the opposite (Figures 1D–F). Thus, CSE may delay IDD progression, whereas CD62E could accelerate it. For the *in vitro* experiments, the reagents used were not cytotoxic at their optimal concentrations after 24 or 48 h treatment (Supplementary Figures 1D–G). To establish the optimal conditions necessary to induce IDD models *in vitro*, rat NP cells were subjected to various IL-1β concentrations and exposure times. The total CSE and CD62E protein levels were significantly altered after 24 h of 5 ng/mL IL-1β treatment (Figures 1G–L). Similar trends were found for TNF-α, which may have a similar proinflammatory effect on NP cells (Supplementary Figures 1A–C). Therefore, the NP cells were exposed to 5 ng/mL of proinflammatory factors for 24 h to induce the IDD model for the subsequent experiments.

### Intervertebral Disc Degeneration Promotes Microvascular Invasion and Angiogenesis

**FIGURE 2:**
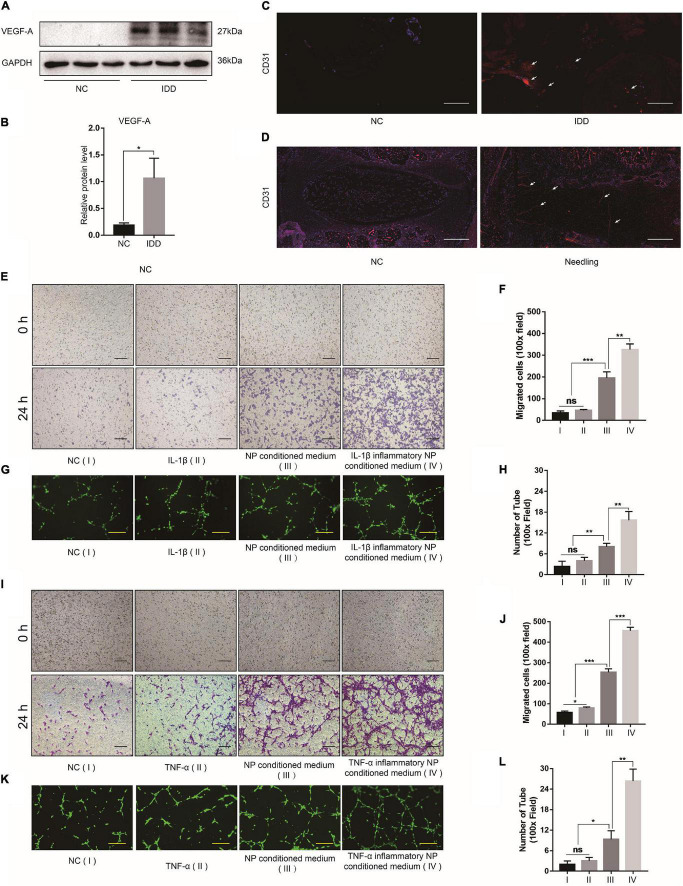
Effects of degenerated NP cells on blood vessel function. **(A)** Representative western blot images of VEGF-A in human NP tissue and **(B)** its quantitative analysis. **(C)** CD31 IF images of human and **(D)** rat NP tissues. **(E)** Bright-field images of migrated EPCs after incubation with IL-1β and **(F)** quantification by ImageJ. **(G)** Calcein-AM IF images of EPC tube formation assay after incubation with IL-1β and **(H)** quantification by ImageJ. **(I)** Bright-field images of migrated EPCs after incubation with TNF-α and **(J)** quantification by ImageJ. **(K)** Calcein-AM IF images of EPC tube formation assay after incubation with TNF-α and **(L)** quantification by ImageJ. Black scale bar: 100 μm. Yellow scale bar: 200 μm. White scale bar: 500 μm.

Western blot indicated that the NP tissues of the IDD patients presented with upregulated VEGF-A compared with those of the NC group (Figures 2A,B). The IF images showed that the blood vessel marker CD31 was also upregulated in the NP tissues of patients with IDD as well as those of the IDD animal model (Figures 2C,D). The *in vivo* assay confirmed that degenerative NP microenvironment may lead to microvascular invasion, angiogenesis, and IDD exacerbation. To confirm the effects of NP cell degeneration on blood vessels, EPCs were cultured for a vascular function assay. The NP cell supernatant was collected and used as NP conditioned (DMEM/F12) medium and as inflammatory NP conditioned (DMEM/F12 with IL-1β or TNF-α) medium. The EPCs were incubated with DMEM/F12, DMEM/F12 with IL-1β, NP conditioned medium, or IL-1β inflammatory NP conditioned medium. Tube formation and transwell migration assays were conducted to evaluate EPC angiogenesis and migration ability. EPCs treated with inflammatory NP conditioned medium exhibited the activation of vascular function (Figures 2E–H). This phenomenon might account for the microvascular invasion and angiogenesis characteristic of IDD progression. Moreover, a similar tendency was observed in EPCs treated with TNF-α inflammatory NP conditioned medium (Figures 2I–L).

### NF-κB Aggravates Intervertebral Disc Degeneration by Promoting Inflammation and Extracellular Matrix Decomposition

**FIGURE 3:**
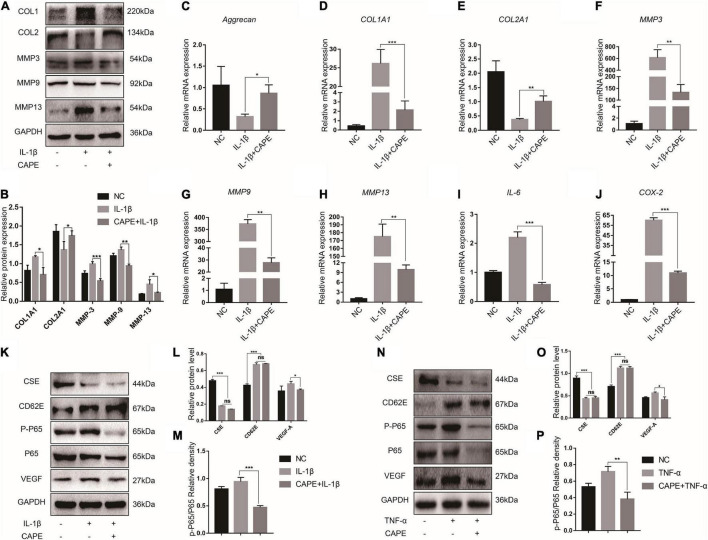
NF-κB function in IDD. **(A)** Western blot analysis of MMPs and ECM components and **(B)** quantification. **(C–J)** RT-PCR measurement of mRNA levels of ECM-encoding genes, ECM-degrading genes, and secreted inflammatory factors. **(K)** Representative western blot images of CSE, CD62E, P65, p-P65, and VEGF-A and **(L,M)** quantification (IL-1β). **(N)** Representative western blot images of CSE, CD62E, P65, p-P65, and VEGF-A and **(O,P)** quantification (TNF-α).

The NF-κB pathway is one of the principle mechanisms associated with inflammation. To explore the effects of the inflammatory microenvironment on NP cells, we evaluated the relative differences in the expression of the major ECM encoding and degrading genes and the secreted inflammatory factors after the proinflammatory factor IL-1β and the NF-κB inhibitor CAPE treatment. The MMP-3/9/13 and COL1A1 protein expression levels increased after IL-1β exposure and decreased after CAPE treatment. However, COL2A1 expression displayed the opposite trend in response to IL-1β and CAPE (Figures 3A,B). Similar results were obtained for MMP-3/9/13, COL1A1, and COL2A1 mRNA expression (Figures 3C–H). NP cells stimulated by IL-1β secreted the proinflammatory factors IL-6 and COX-2. Nevertheless, these effects were inhibited by CAPE (Figures 3I,J). Hence, blocking the NF-κB pathway may inhibit inflammation and ECM decomposition in NP cells. We explored the regulatory relationships among the NF-κB pathway-related proteins (P65 and p-P65), CSE, CD62E, and VEGF-A. Figures 3K–P indicate that CD62E, p-P65, and VEGF-A were significantly upregulated in response to IL-1β and TNF-α, whereas CSE was notably downregulated. Activated p-P65 and VEGF-A were inhibited by CAPE. However, CSE and CD62E had no obvious impact after blocking the NF-κB pathway (CAPE). The regulatory relationships among CSE, CD62E, and the NF-κB pathway require further investigation.

### Blocking CD62E Attenuated the P65 Pathway

**FIGURE 4:**
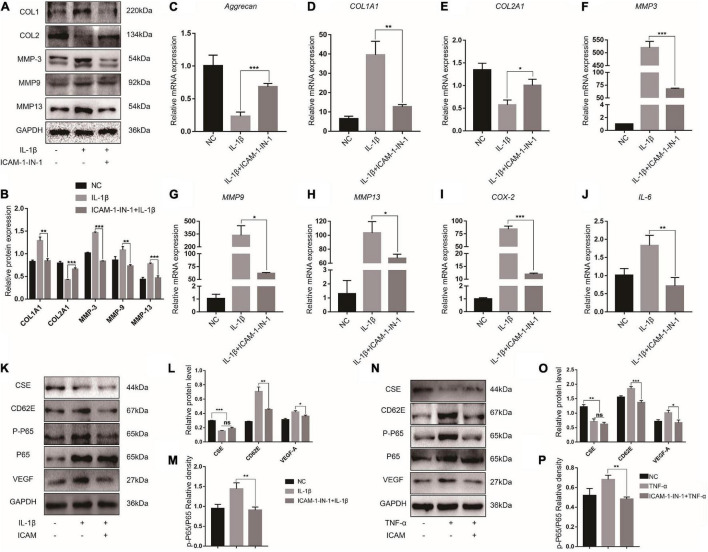
Blocking CD62E influences inflammatory NP cells. **(A)** Representative western blot images and **(B)** quantification of effect of blocking CD62E in NP cells. **(C–J)** RT-PCR measurement of mRNA levels of ECM-encoding genes, ECM-degrading genes, and secreted inflammatory factors. **(K)** Representative western blot images of CSE, CD62E, P65, p-P65, and VEGF-A and **(L,M)** quantification (IL-1β). **(N)** Representative western blot images of CSE, CD62E, P65, p-P65, and VEGF-A and **(O,P)** quantification (TNF-α).

To investigate the effects of CD62E on IL-1β-induced NP destruction, CD62E was blocked with ICAM-1-IN-1. After the NP cells responded to proinflammatory stimuli, CD62E inhibition downregulated MMP-3/9/13 and COL1A1 but upregulated COL2A1 at the mRNA expression and protein levels (Figures 4A–H). Similarly, the proinflammatory factor genes were downregulated (Figures 4I,J). To explore the effects of CD62E on the inflammation pathway, CSE, p-P65, and VEGF-A were detected by western blotting (Figures 4K–P). After the treatment, CSE that had been deactivated by IL-1β or TNF-α could not be reactivated by CD62E inhibition. In contrast, CD62E, p-P65, and VEGF-A upregulation was suppressed by ICAM-1-IN-1. Our results indicated that CD62E may be regulated by CSE and affects the P65 pathway in NP cells.

### Effects of Cystathionine-γ-Lyase on Nucleus Pulposus Cell Inflammation and Extracellular Matrix by CD62/P65 Inhibition

**FIGURE 5:**
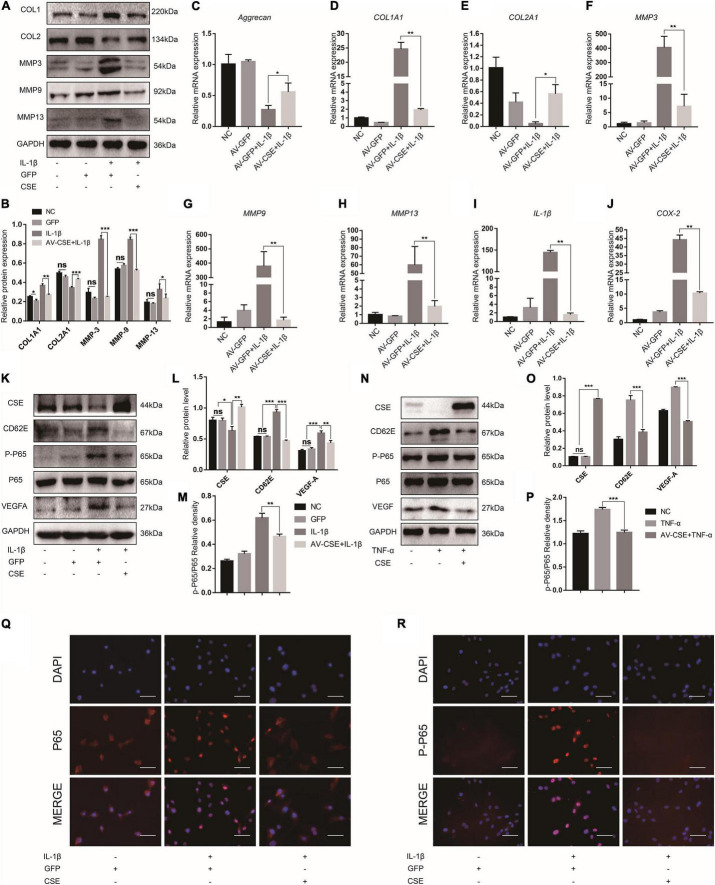
Cystathionine-γ-lyase overexpression inhibits CD62E and NF-κB signaling pathways in NP cells. **(A)** Representative western blot images and **(B)** quantification of effect of CSE overexpression in NP cells. **(C–J)** RT-PCR measurement of mRNA levels of ECM-encoding genes, ECM-degrading genes, and secreted inflammatory factors. **(K)** Representative western blot images of CSE, CD62E, P65, p-P65, and VEGF-A and **(L,M)** quantification (IL-1β). **(N)** Representative western blot images of CSE, CD62E, P65, p-P65, and VEGF-A and **(O,P)** quantification (TNF-α). **(Q)** IF analysis of P65 and **(R)** p-P65 after IL-1β treatment. White scale bar: 50 μm.

**FIGURE 6:**
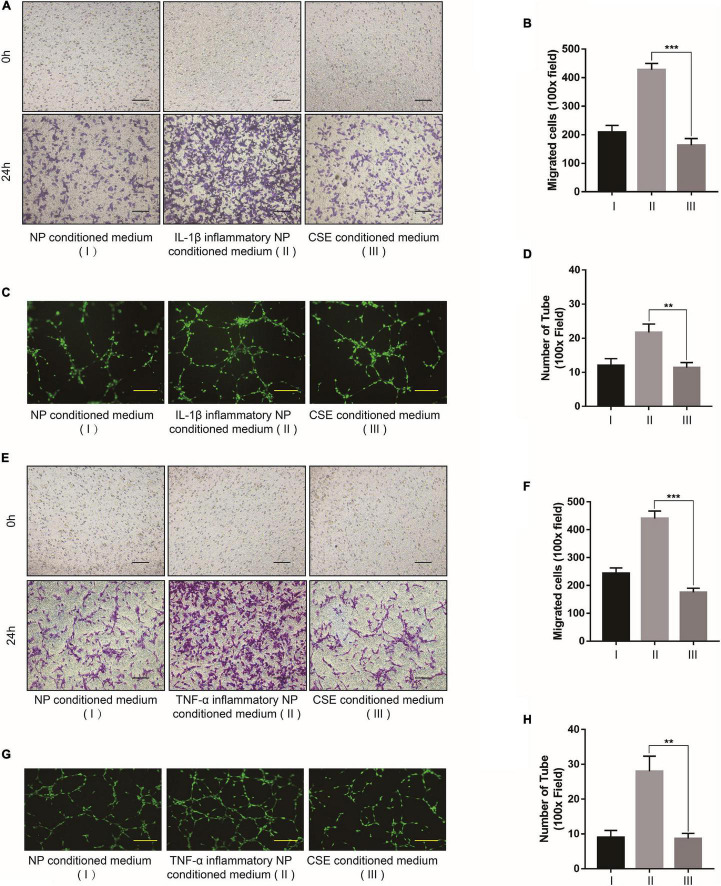
Cystathionine-γ-lyase overexpression in NP cell inhibits vascular function. **(A)** Bright-field images of migrated EPCs after incubation with IL-1β and **(B)** quantification by ImageJ. **(C)** Calcein-AM IF images of EPC tube formation assay after incubation with IL-1β and **(D)** quantification by ImageJ. **(E)** Bright-field images of migrated EPCs after incubation with TNF-α and **(F)** quantification by ImageJ. **(G)** Calcein-AM IF images of EPC tube formation assay after incubation with TNF-α and **(H)** quantification by ImageJ. Black scale bar: 100 μm. Yellow scale bar: 200 μm.

To examine the anti-inflammatory effect of CSE, we overexpressed it in NP cells *via* AV-CSE and confirmed successful transfection (MOI = 100) (Supplementary Figure 2A). CSE overexpression exhibited good therapeutic efficacy against inflammation and ECM degeneration. It downregulated MMP-3/9/13 and COL1A1, upregulated COL2A1, and downregulated proinflammatory factor gene levels (Figures 5A–J). While CD62E, p-P65, and VEGF-A levels were promoted by inflammatory irritation, they were significantly inhibited by AV-CSE treatment (Figures 5K–P). Hence, the CD62E and NF-κB pathways in the NP cells might be regulated by CSE. To assess the association between CSE and P65, we detected P65 activation in NP cells induced by IL-1β or TNF-α. P65 is a nuclear factor that exerts transcriptional activity by translocating to the nucleus and phosphorylating to induce its downstream genes expression. IF images showed that P65 is normally distributed in the cytoplasm rather than the nucleus (NC group) and translocated to the nucleus only after inflammatory irritation. Similarly, phosphorylated P65 was mainly detected in the nucleus after inflammatory stimulation. However, nuclear translocation and phosphorylation of P65 induced by inflammatory stimulation were significantly inhibited by AV-CSE (Figures 5Q,R and Supplementary Figure 3). In addition, the suppression effect of vascular function by AV-CSE was also verified. Figure 6 shows that EPCs cultured in AV-CSE NP conditioned medium (DMEM/F12 with IL-1 *i*th IL-l-CSE) presented with reduced angiogenesis and migration compared with EPCs cultured in the inflammatory NP-conditioned medium. Thus, CSE could effectively regulate inflammation and vascular function in IDD progression.

### Cystathionine-γ-Lyase Overexpression Alleviates Intervertebral Disc Degeneration Degradation in a Rat Intervertebral Disc Degeneration Model

**FIGURE 7:**
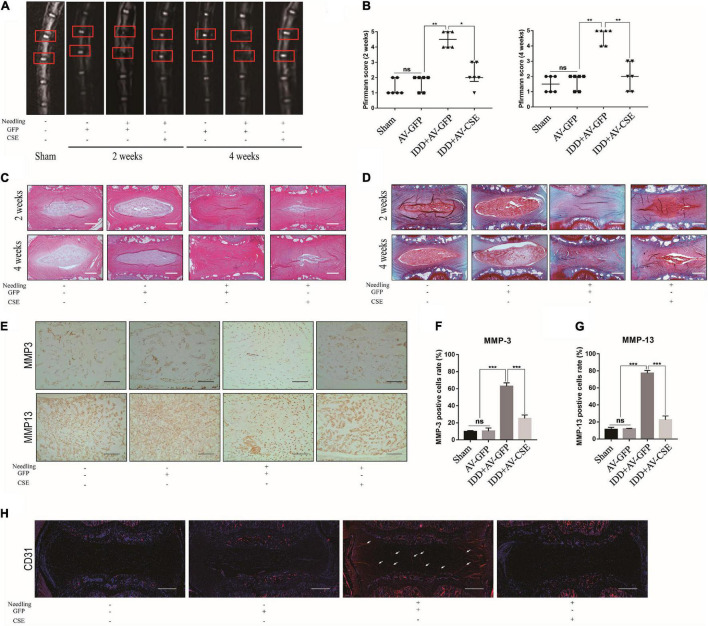
Effects of AV-CSE on rat IDD model. **(A)** T2-weighted MRI images of rat tail at 2 and 4 weeks post-surgery (red boxes: needle-puncture disc locations). **(B)** Pfirrmann MRI scores (*n* = 6). **(C)** Representative H&E and **(D)** Safranin O/Fast Green staining images. **(E)** IHC images of MMP-3 and MMP-13 and **(F,G)** quantification. **(H)** CD31 IF images. Black scale bar: 100 μm. White scale bar: 500 μm.

To investigate the roles of CSE in IDD pathogenesis *in vivo*, a rat IDD model was induced by disc puncture and subjected to the foregoing treatments. After the surgical procedure, protein-level CSE overexpression in IVD was verified by western blot and IHC (Supplementary Figures 2C–E). MRI scans of the model IVD were performed at 2 and 4 weeks postoperatively. MRI images of the IDD + AV-GFP group revealed degenerative changes in the IVD and altered coccygeal intervertebral disc structures after surgery. By contrast, the IDD + AV-CSE group presented with a noticeable amelioration of the IDD (Figures 7A,B). Relative to the Sham group, there were no significant changes in the IVDs injected with AV-GFP alone. Hence, the adenovirus vector injection was not injurious to the NP tissues. H&E and Safranin O/Fast Green staining displayed a range of postoperative morphological changes. Post-surgery ECM reduction and aggravation of fibrosis in NP significantly decreased after the AV-CSE injections (Figures 7C,D). Besides, the expression of MMP-3 and MMP-13 was significantly decreased in the IDD + AV-CSE group compared to the IDD + AV-GFP group (Figures 7E–G). CD31 IF staining showed prominent blood vessel architecture and vascular invasion in the IDD + AV-GFP group NP tissue and suppression of these disease progression symptoms by AV-CSE (Figure 7H). Thus, CSE overexpression in the IVD mitigated inflammation and vascular invasion and impeded IDD progression.

### CD62E Overexpression Aggravates Intervertebral Disc Degeneration Degradation in a Rat Intervertebral Disc Degeneration Model

**FIGURE 8:**
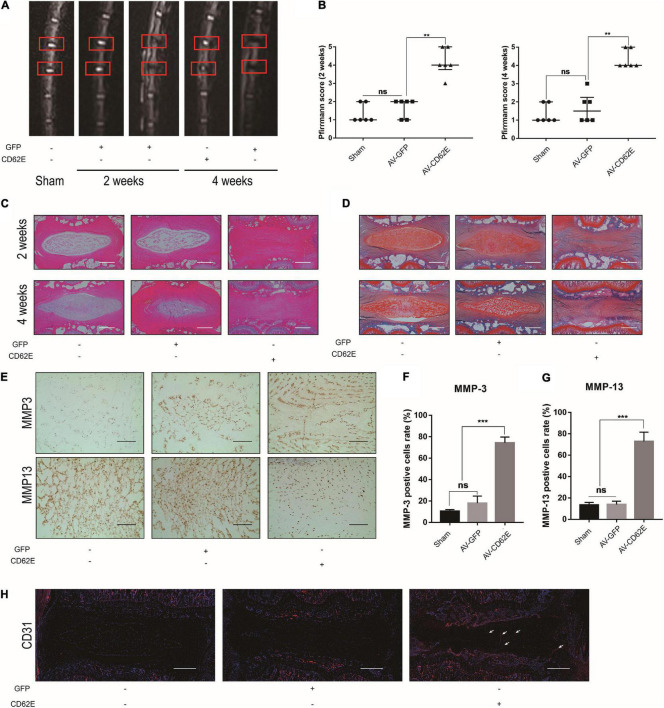
Effects of AV-CD62E on rat IDD model. **(A)** T2-weighted MRI images of rat tail at 2 and 4 weeks post-surgery (red boxes: needle-puncture disc locations). **(B)** Pfirrmann MRI scores (*n* = 6). **(C)** Representative H&E and **(D)** Safranin O/Fast Green staining images. **(E)** IHC images of MMP-3 and MMP-13 and **(F,G)** quantification. **(H)** CD31 IF images. Black scale bar: 100 μm. White scale bar: 500 μm.

CD62E activation promoted inflammation in NP cells. To examine the roles of CD62E in IDD pathogenesis *in vivo*, we subjected rat’s NP tissue to Sham, AV-GFP, or AV-CD62E treatment. The MRI images in Figures 8A,B show that the vertebral discs in the AV-CD62E group were significantly degenerated compared to the others. H&E and Safranin O/Fast Green staining disclosed IDD deterioration in rats administered AV-CD62E by IVD injection (Figures 8C,D). Moreover, MMP-3 and MMP-13 were strongly upregulated in the AV-CD62E group, which presented with ECM decomposition and exacerbation of fibrosis (Figures 8E–G). CD31 IF staining revealed that blood vessels were far more distributed in the AV-CD62E group NP tissue than the AV-GFP or Sham group (Figure 8H). The foregoing results demonstrated that CD62E may aggravate IDD.

## Discussion

Conservative and drug therapies may be preferable in early-onset IDD. However, clinical trials have shown that surgery is an efficacious method of treating this disorder (Wu et al., 2020). The foregoing therapeutic measures have numerous limitations and side effects in the long term (Guterl et al., 2013). Furthermore, they only focus on alleviating the clinical symptoms of IDD rather than retarding IVD degeneration (van Uden et al., 2017). Hence, the pathological processes and mechanisms of IDD have become research hotspots and are potential targets for IDD drug therapy. To the best of our knowledge, the present study is the first to report that CSE and CD62E regulate NF-κB (P65) and VEGF-A expression and modulate IDD progression.

Intervertebral disc inflammation is a major molecular mechanism in IDD progression. Several factors contribute to the inflammatory environment of the IVD, including inflammatory factor stimulation (Podichetty, 2007; Wang et al., 2020), inflammatory macrophage activity (Vizcaíno Revés et al., 2020; Li et al., 2021), abnormal glucose metabolism (Qi et al., 2019; Shan et al., 2019; Silagi et al., 2020), and oxidative stress (Feng et al., 2017a, b; Kang et al., 2020), and so on. In the present study, IL-1β and TNF-α were used to induce NP cells and simulate the inflammatory disc environment (Wang et al., 2017, 2020). The regulatory relationships among CSE/CD62E/NF-κB (P65)/VEGF-A were then demonstrated at the protein levels under different intervention conditions (CSE overexpression, CD62, and NF-?B inhibition). CSE is a key protein that may inhibit the activation of downstream inflammatory pathways and, by extension, alleviate IVD inflammation. We also measured gene- and protein-level expression of MMPs, COL I, and COL II which reflect NP cell anabolism, under various conditions. NP cell ECM anabolism and function are recovered by upregulating CSE and inhibiting CD62/NF-κB (P65). This discovery confirmed that the foregoing pathway mitigates IVD inflammation and recovers the physiological function of NP cells. The MRI and IHC outcomes of our *in vivo* experiments validated our hypothesis. The deterioration of the rat disc IDD model was significantly ameliorated by the AV-CSE treatment compared to the IDD + AV-GFP group. By contrast, AV-CD62E administration exacerbated IDD progression. Hence, CSE could effectively treat IDD, whereas CD62E might initiate the inflammatory response.

Microvessel invasion in IVD is also a critical factor in IDD progression (Sakai and Grad, 2015; Oichi et al., 2020) and may be associated with intervertebral disc herniation and VEGF expression (Kokubo et al., 2008). Blood vessel emergence in the NP tissue might recruit proinflammatory cells and constitutes an auto-immune response of the NP (Sun et al., 2020). It also aggravates oxidative stress and accelerates IVD degeneration. Here, we performed several experiments on EPCs to determine whether CSE protein regulates vascular function. The results of the *in vitro* assays revealed that CSE effectively downregulated VEGF-A in NP cells and inhibited angiogenesis and migration in EPCs. The *in vivo* assays disclosed that the vascular function marker CD31 was significantly downregulated in the IDD model rat NP tissue after CSE adenovirus treatment. Therefore, CSE might be a therapeutic target for inhibiting vascular invasion in IDD.

There were certain limitations to the present research. In our previous study, we proposed that the presence of CSE was strongly associated with suppression of the CD62E/NF-κB (P65)/VEGF-A pathway. However, the specific mechanisms and target locations of these pathways in IDD pathogenesis and the involvement of CSE in other IDD-related signaling pathways remain to be established. IDD-associated research has achieved many impressive results. Nevertheless, it is difficult to intervene in clinical IVD cases and the practical application of CSE therapy for IDD might be challenging.

**FIGURE 9:**
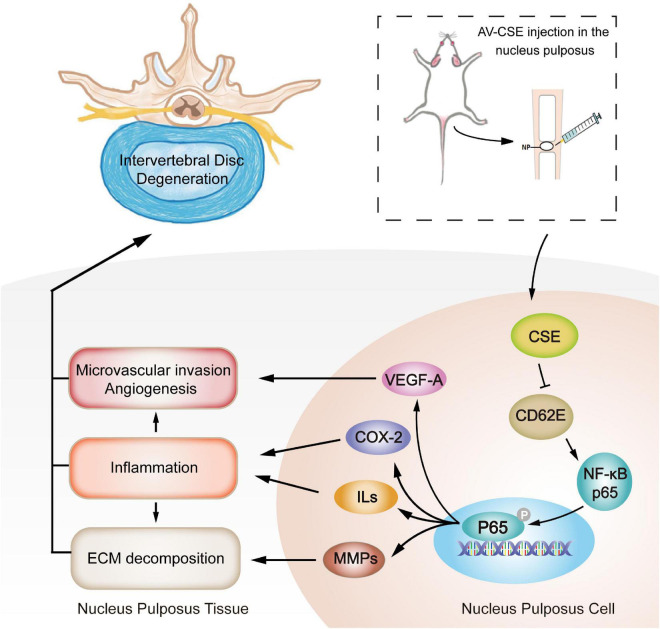
Schematic illustration of mitigation of inflammation, microvascular invasion, and ECM decomposition in IDD progression mediated by AV-CSE treatment.

Despite these difficulties, it is undeniable that our current research on the mechanism of IDD has laid the foundation for the development and optimization of efficacious, innovative treatments for IDD degenerative disease. In this study, CSE exhibited potent anti-inflammatory efficacy, whereas CD62E activated the inflammatory environment. Subsequent *in vitro* and *in vivo* assays showed that CSE overexpression inhibited the CD62E-NF-κB (P65) pathway activation and downstream inflammatory factor secretion (VEGF-A, ILs, COX-2, and MMPs). These effects improved IDD by reducing inflammatory irritation, ECM decomposition, and microvascular invasion in the NP tissue (Figure 9). In conclusion, CSE protein simultaneously downregulated CD62E and blocked the NF-κB (P65) pathways in IVD. Hence, it may impede the progression of IDD. The encouraging results of this pilot study indicate that the pathways regulated by CSE are promising candidate targets for clinical IDD therapy.

## Data Availability Statement

Publicly available datasets were analyzed in this study. These data can be found here: microarray dataset (accession no. GSE34095) was obtained from the GEO database (https://www.ncbi.nlm.nih.gov/gds).

## Ethics Statement

The animal study was reviewed and approved by the Ethics Committee on Animal Experimentation of Tongji Hospital, Huazhong University of Science and Technology. Written informed consent was obtained from the individual(s) and minor(s)’ legal guardian/next of kin for the publication of any potentially identifiable images or data included in this article.

## Author Contributions

HW and HF conceived and designed this study. HaoX and KW performed most of the experiments and wrote the manuscript. JT and YC performed the bioinformatics analysis and revised and proofread the manuscript. YH and YD fed the experimental animals and helped with IDD model establishment. HuaX and XB performed the statistical analysis on data. HuiX performed the MRI scan and analyzed related data. All authors provided editorial comments and agreed to be accountable for the content of the work.

## Conflict of Interest

The authors declare that the research was conducted in the absence of any commercial or financial relationships that could be construed as a potential conflict of interest.

## Publisher’s Note

All claims expressed in this article are solely those of the authors and do not necessarily represent those of their affiliated organizations, or those of the publisher, the editors and the reviewers. Any product that may be evaluated in this article, or claim that may be made by its manufacturer, is not guaranteed or endorsed by the publisher.

## Footnotes

1
https://www.ncbi.nlm.nih.gov/gds
