# Supplementary material for: Reducing Inflammation and Vascular Invasion in Intervertebral Disc Degeneration via Cystathionine-γ-Lyase Inhibitory Effect on E-Selectin
Source: Front Cell Dev Biol. 2021 Nov 15;9:741046. doi: 10.3389/fcell.2021.741046 (PMC8634256; doi:10.3389/fcell.2021.741046)
Supplement: Supplementary file 1 [file Table_1.DOCX]

Supplementary Material


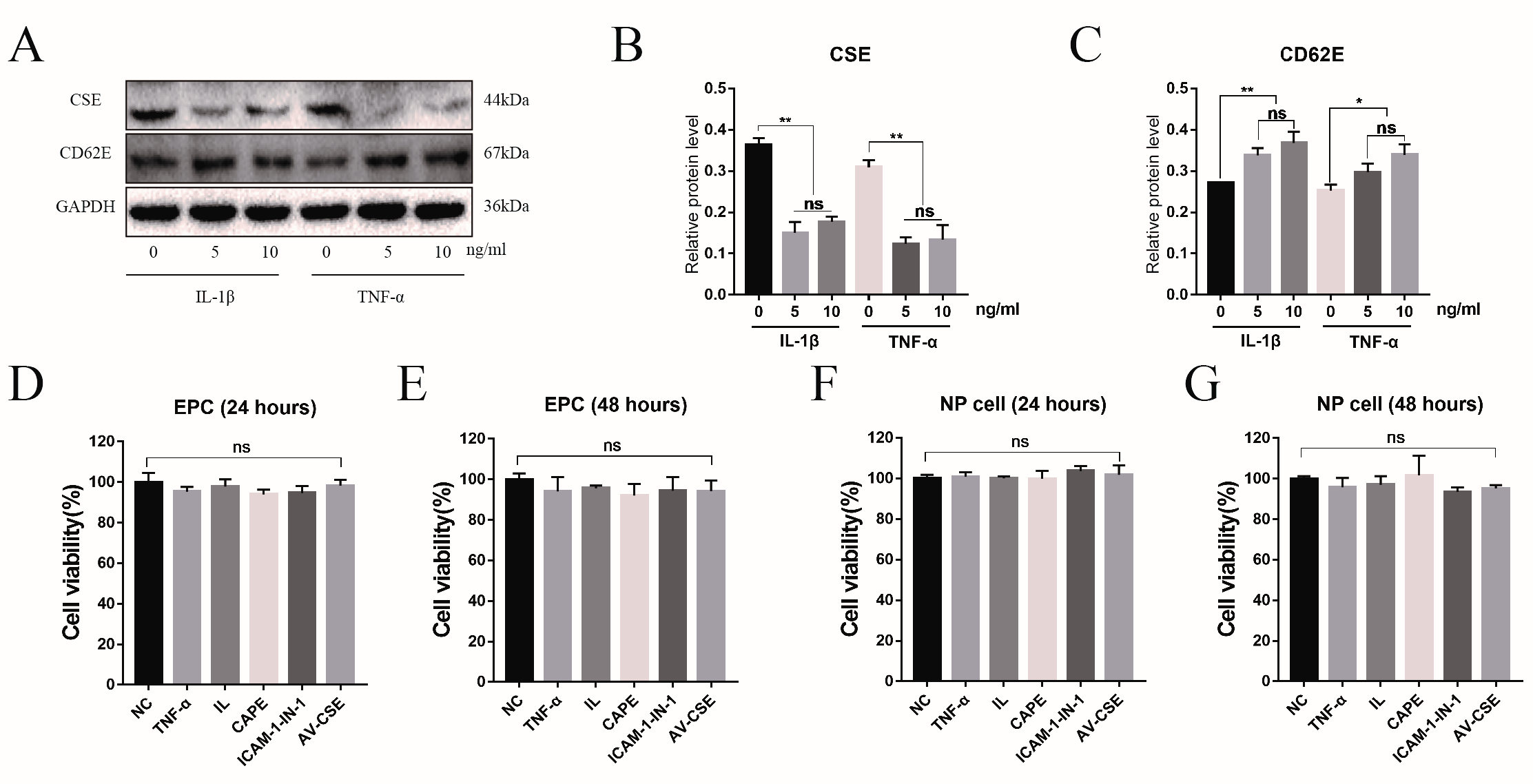


**Supplementary Figure 1.** Supplementary figures of a series of *in vitro* experiments. **(A)** Representative western blot and **(B,C)** its quantification of the CSE and CD62E under IL-1β or TNF-α treatment(5,10 ng/ml). The cell viability on EPC for **(D)** 24 hours or **(E)** 48 hours and on NP cell for **(F)** 24 hours or **(G)** 48 hours were evaluated by CCK-8 assay.


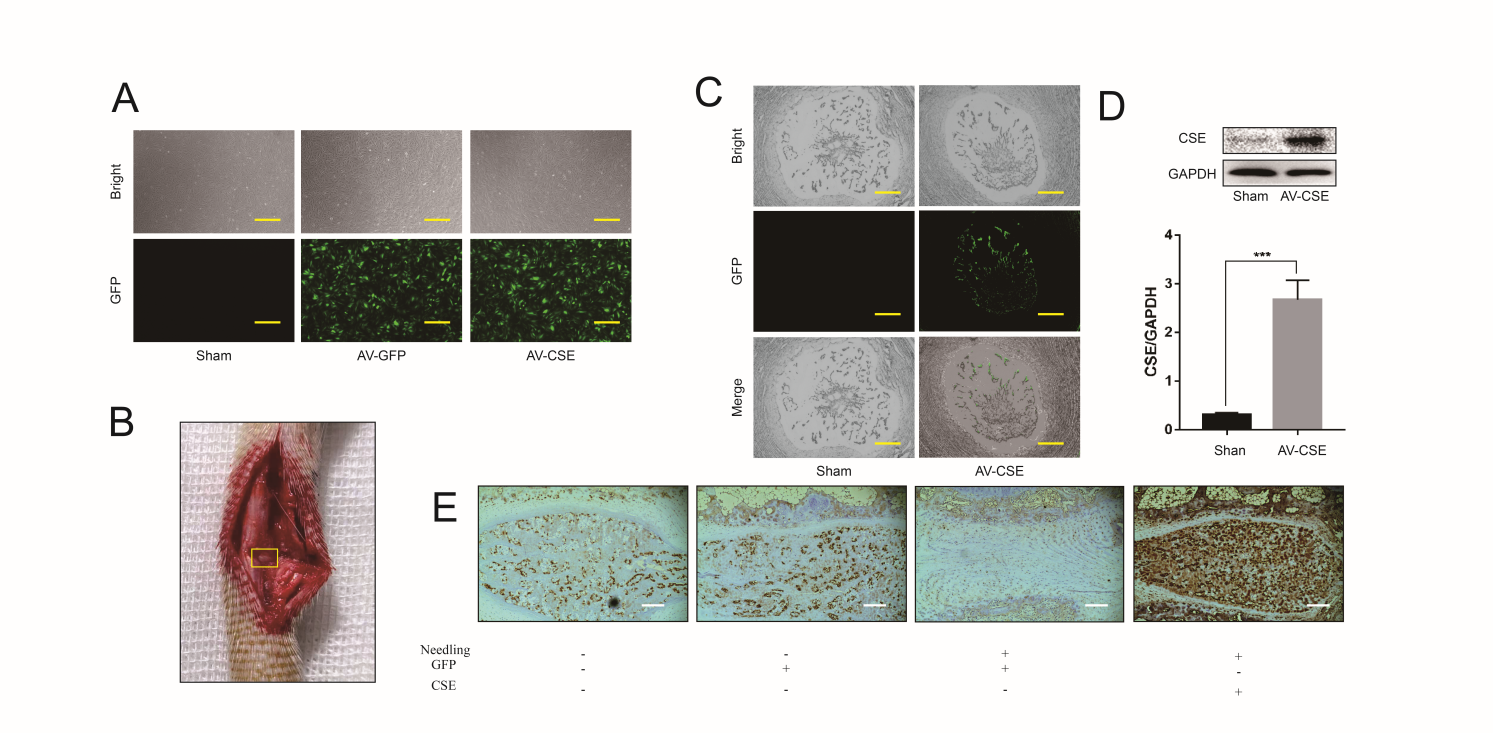


**Supplementary Figure 2.** AV-CSE overexpression verification *in vitro* and *in vivo*. **(A)** Fluorescence images of NP cells transfected with adenovirus. **(B)** Schematic diagram of rat's IVD exposure in animal experiments. 4 weeks after operation, **(C)** CSE immunofluorescence image, **(D)** CSE protein expression and **(E)** CSE IHC images of rat IVD. Yellow scale bar: 500 μm, white scale bar: 200 μm.

**
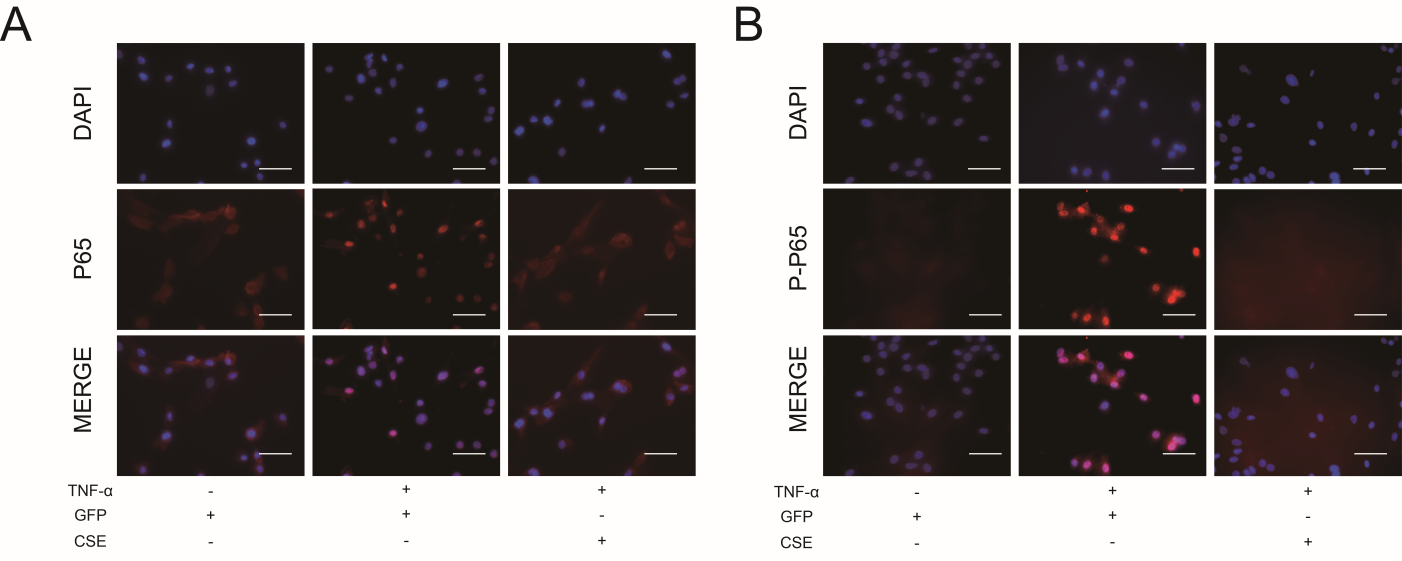
**

**Supplementary Figure 3.** IF analysis of **(A)** P65 and **(B)** p-P65 after TNF-α treatment. White scale bar: 50 μm.

**Table 1.** RT-PCR primers used for gene expression analysis.

| Gene name | Forward | Reverse |
| --- | --- | --- |
| *GAPDH* | GGTGAAGGTCGGTGTGAACG | CTCGCTCCTGGAAGATGGTG |
| *MMP-3* | GCTCATCCTACCCATTGCAT | GCTTCCCTGTCATCTTCAGC |
| *MMP-9* | TCCTTGCAATGTGGATGTTT | CGTCCTTGAAGAAATGCAGA |
| *MMP-13* | CAAGCAGCTCCAAAGGCTAC | TGGCTTTTGCCAGTGTAGGT |
| *COL1A1* | CGTGGAAACCTGATGTATGC | GGTTGGGACAGTCCAAGTCT |
| *COL2A1* | CGAGGCAGACAGTACCTTG | TGCTCTCGATCTGGTTGTTC |
| *Aggrecan* | CTTCCCAACTATCCAGCCAT | TCACACCGATAGATCCCAGA |
| *COX-2* | CTCAGCCATGCAGCAAATCC | GGGTGGGCTTCAGCAGTAAT |
| *IL-1β* | CAGCTTTCGACAGTGAGGAGA | TTGTCGAGATGCTGCTGTGA |
| *IL-6* | AGAGACTTCCAGCCAGTTGC | TGCCATTGCACAACTCTTTTC |
